# Supplementary material for: Opposing effects of selectivity and invariance in peripheral vision
Source: Nat Commun. 2021 Jul 28;12:4597. doi: 10.1038/s41467-021-24880-5 (PMC8319169; doi:10.1038/s41467-021-24880-5)
Supplement: Supplementary file 1 — Supplementary Information [file 41467_2021_24880_MOESM1_ESM.pdf]

Supplementary Information

Opposing effects of selectivity and invariance in peripheral vision

Corey M. Ziemba & Eero P. Simoncelli

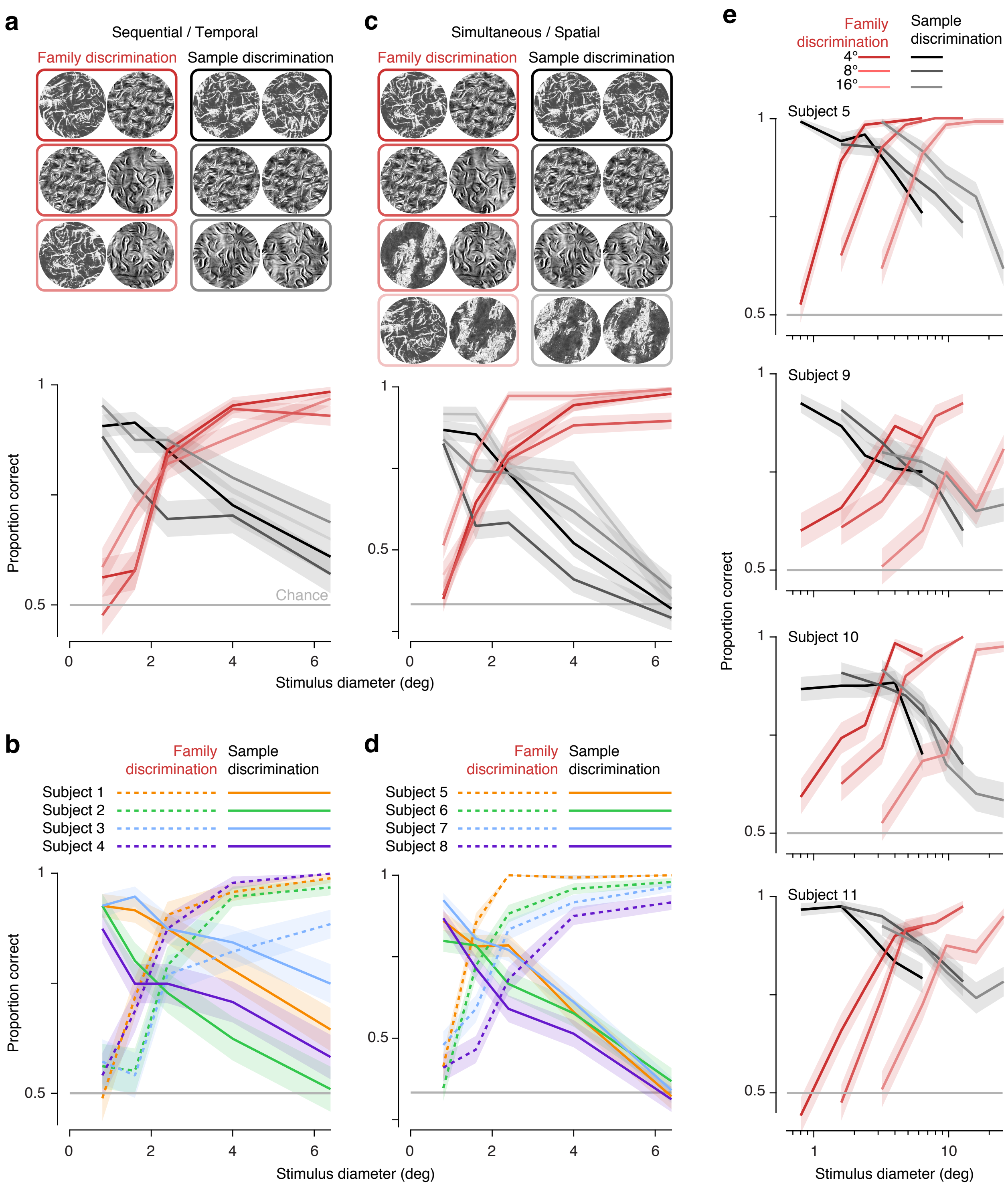

Supplementary Fig. 1 Discrimination behavior across texture stimuli and subjects. a Average proportion correct for 4 subjects performing the sequentially presented task. Subjects discriminated 3 different pairs of texture families (red), or samples drawn from within 3 different families (black). Performance in each of these 6 conditions is plotted separately. **b** Performance on the same sequentially presented task, averaged across all stimulus conditions, but plotted separately by observer. **c-d** Same as (**a-b**) but for the simultaneous version of the task. Subjects saw 4 different pairs of texture families (red), or samples drawn from within 4 different families (black) because of the extra time afforded by the short trial duration of the simultaneous task. **e** Proportion correct for individual subjects performing sequential family and sample discrimination at different eccentricities. Performance is averaged over the 3 family and sample discrimination stimuli used in the sequential version of the task as in (**a**). Red lines indicate family and black lines indicate sample discrimination as in Figure 4 with lighter colored lines indicating larger eccentricities. Subject 5 was an author. Shaded regions indicate mean ± SEM across trials in all panels. Source data are provided as a Source Data file.

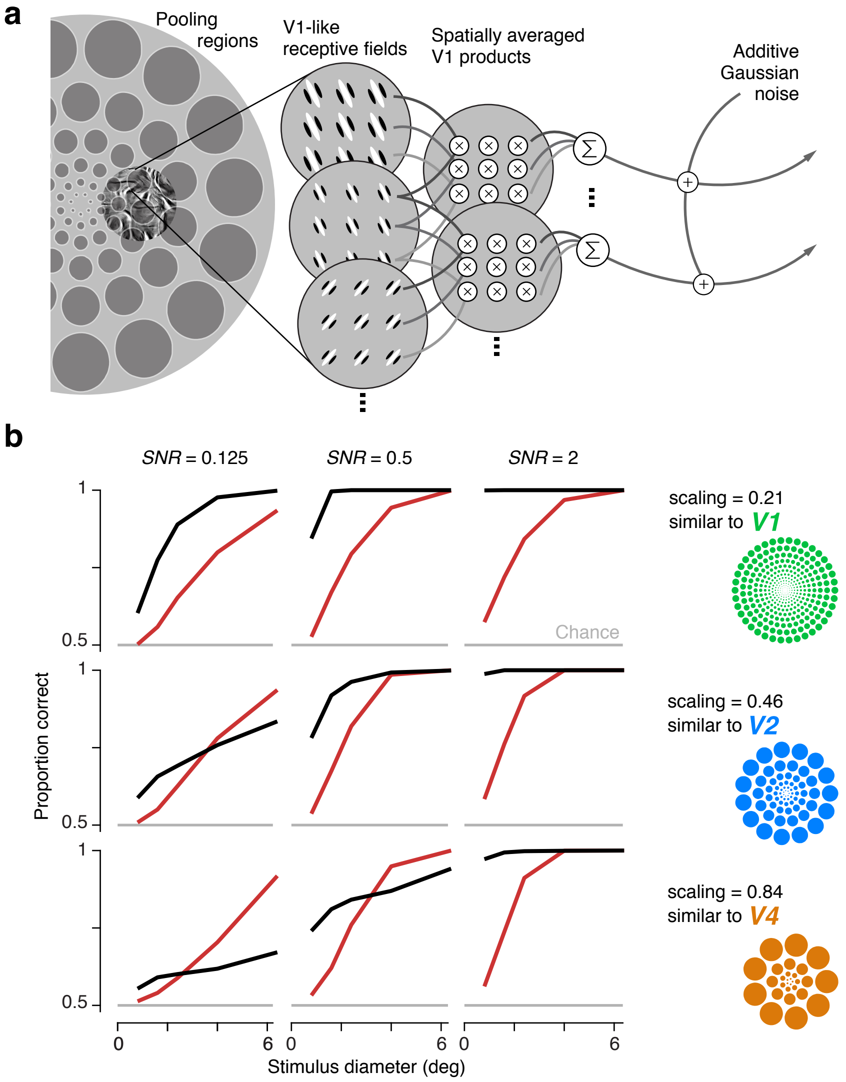

Supplementary Fig. 2 A two-stage observer model without normalization fails to predict opposing perceptual effects. a Model diagram as in Fig. 3a, but without normalization. **b** Performance on family (red) and sample (black) discrimination tasks, as a function of stimulus diameter, for observer models with three different levels of noise and three different scaling values. The average signal-to-noise ratio was computed separately for each scaling by dividing the variance in responses across all stimuli and pooling regions by the variance of the Gaussian noise. Scaling values (stimulus diameter divided by eccentricity) are matched to estimates from visual areas V1, V2, and V4. Source data are provided as a Source Data file.
