# Supplementary material for: Opposing effects of selectivity and invariance in peripheral vision
Source: Nat Commun. 2021 Jul 28;12:4597. doi: 10.1038/s41467-021-24880-5 (PMC8319169; doi:10.1038/s41467-021-24880-5)
Supplement: Supplementary file 3 — Reporting Summary [file 41467_2021_24880_MOESM3_ESM.pdf]

# nature research

Corresponding author(s): Corey M Ziemba

Last updated by author(s): Jun 4, 2021

# Reporting Summary

Nature Research wishes to improve the reproducibility of the work that we publish. This form provides structure for consistency and transparency in reporting. For further information on Nature Research policies, see our Editorial Policies and the Editorial Policy Checklist.

## Statistics

For all statistical analyses, confirm that the following items are present in the figure legend, table legend, main text, or Methods section.

| n/a | Confirmed | |
|---|---|---|
| ☐ | ☒ | The exact sample size ($n$) for each experimental group/condition, given as a discrete number and unit of measurement |
| ☒ | ☐ | A statement on whether measurements were taken from distinct samples or whether the same sample was measured repeatedly |
| ☒ | ☐ | The statistical test(s) used AND whether they are one- or two-sided<br>*Only common tests should be described solely by name; describe more complex techniques in the Methods section.* |
| ☒ | ☐ | A description of all covariates tested |
| ☒ | ☐ | A description of any assumptions or corrections, such as tests of normality and adjustment for multiple comparisons |
| ☐ | ☒ | A full description of the statistical parameters including central tendency (e.g. means) or other basic estimates (e.g. regression coefficient) AND variation (e.g. standard deviation) or associated estimates of uncertainty (e.g. confidence intervals) |
| ☒ | ☐ | For null hypothesis testing, the test statistic (e.g. $F$, $t$, $r$) with confidence intervals, effect sizes, degrees of freedom and $P$ value noted<br>*Give P values as exact values whenever suitable.* |
| ☒ | ☐ | For Bayesian analysis, information on the choice of priors and Markov chain Monte Carlo settings |
| ☒ | ☐ | For hierarchical and complex designs, identification of the appropriate level for tests and full reporting of outcomes |
| ☒ | ☐ | Estimates of effect sizes (e.g. Cohen's $d$, Pearson's $r$), indicating how they were calculated |

*Our web collection on statistics for biologists contains articles on many of the points above.*

## Software and code

Policy information about availability of computer code

| | |
|---|---|
| Data collection | We used MGL v1.0 to collect psychophysical data. The custom program is available in a public repository (https://doi.org/10.17605/OSF.IO/GFEPH). Stimuli were generated using texture analysis/synthesis code v1.0 available at www.cns.nyu.edu/~lcv/texture/ |
| Data analysis | All data analyses and simulations were performed using Matlab v9.4.0.813654 (R2018a), including modification of the visual metamers code v1.0 available at https://github.com/freeman-lab/metamers. All code used for analyses and simulations is available in a public repository (https://doi.org/10.17605/OSF.IO/GFEPH). |

For manuscripts utilizing custom algorithms or software that are central to the research but not yet described in published literature, software must be made available to editors and reviewers. We strongly encourage code deposition in a community repository (e.g. GitHub). See the Nature Research guidelines for submitting code & software for further information.

## Data

Policy information about availability of data

All manuscripts must include a data availability statement. This statement should provide the following information, where applicable:

- Accession codes, unique identifiers, or web links for publicly available datasets
- A list of figures that have associated raw data
- A description of any restrictions on data availability

The psychophysical data that support the findings of this study are available in a public repository (https://doi.org/10.17605/OSF.IO/GFEPH).

# Field-specific reporting

Please select the one below that is the best fit for your research. If you are not sure, read the appropriate sections before making your selection.

☒ Life sciences ☐ Behavioural & social sciences ☐ Ecological, evolutionary & environmental sciences

For a reference copy of the document with all sections, see nature.com/documents/nr-reporting-summary-flat.pdf

# Life sciences study design

All studies must disclose on these points even when the disclosure is negative.

| | |
|---|---|
| Sample size | 4 subjects performed each experiment. Since an author participated as a subject, we considered replicating our findings more than twice in naive subjects sufficient to demonstrate the strength and generality of the main effect. When we observed no meaningful inter-subject variation in our first experiment, we continued with this number of subjects for the remaining experiments. |
| Data exclusions | No data were excluded. |
| Replication | The main effect was replicated independently in 4 subjects in experiment 1. This effect was further replicated in another 4 subjects under different stimulus presentation conditions in experiment 2. Finally, in experiment 3, 1 subject replicated their performance from experiment 2, and the effect was independently replicated in a further 3 subjects. All attempts at replication were successful. |
| Randomization | Participants were not grouped and hence no randomization was performed. |
| Blinding | Data collection was performed automatically, conditions were randomized in their presentation, and an experimenter was not present with participants during experiments, so experimenters were effectively blind to the outcome except when participating as a subject. |

# Reporting for specific materials, systems and methods

We require information from authors about some types of materials, experimental systems and methods used in many studies. Here, indicate whether each material, system or method listed is relevant to your study. If you are not sure if a list item applies to your research, read the appropriate section before selecting a response.

## Materials & experimental systems

| n/a | Involved in the study | |
|---|---|---|
| ☒ | ☐ | Antibodies |
| ☒ | ☐ | Eukaryotic cell lines |
| ☒ | ☐ | Palaeontology and archaeology |
| ☒ | ☐ | Animals and other organisms |
| ☐ | ☒ | Human research participants |
| ☒ | ☐ | Clinical data |
| ☒ | ☐ | Dual use research of concern |

## Methods

| n/a | Involved in the study | |
|---|---|---|
| ☒ | ☐ | ChIP-seq |
| ☒ | ☐ | Flow cytometry |
| ☒ | ☐ | MRI-based neuroimaging |

## Human research participants

Policy information about studies involving human research participants

| | |
|---|---|
| Population characteristics | 11 Subjects participated (ages 19-30, 7 female, 4 male). |
| Recruitment | We recruited through posting fliers in hallways and common areas of the department building. One subject was an author. The results are unlikely to be affected by any selection bias given the task was a simple repeated visual discrimination. |
| Ethics oversight | Experimental procedures for human subjects were approved by the Institutional Review Board at New York University. All subjects provided written informed consent. |

Note that full information on the approval of the study protocol must also be provided in the manuscript.
